# Can cardiothoracic surgeons provide a safe and effective thoracic surgery service?

**DOI:** 10.1186/1749-8090-10-S1-A175

**Published:** 2015-12-16

**Authors:** Rajani Rajnish, David Rose, John Massey, Royan Richards, Mohamad N Bittar, Andrew J Duncan, Joseph Zacharias, Manoj Purohit

**Affiliations:** 1 Department of Cardiothoracic Surgery Blackpool Victoria Hospital Whinney Heys Road, Blackpool, Lancashire, FY3 8NR, UK

## Background/Introduction

There is a trend to separate Thoracic surgery from the Cardiothoracic surgical divisions. One of the main persuasive argument is the evidence in support of increased resection rate and effective delivery of thoracic surgical services. In such context we reviewed the outcome of thoracic surgical practice in our region where a cardiothoracic surgeon's service was standard.

## Aims/Objectives

To establish via our limited study that Cardiothoracic Surgeons can provide a safe, effective and result oriented thoracic surgery survive.

## Method

Patients were prospectively added, retrospectively analysed for the period from April 2005 to April 2014. Data were collected from PATs database, Cyber lab and Lung cancer MDT database. Data were analysed using Student's t-test, Excel and Kaplan-Meier statistical analysis software.

## Results

A Total of 1057 primary lung cancer patients were operated on, 831 (75.39%) Lobectomy, 147 (13.86%) Wedge resection and 79 (7.45%) Pneumonectomy.

119 (8.9%) patient found to have N2 Positive disease of which 53 (5%) received Chemotherapy and 6 (0.56%) had radiotherapy.

Our in hospital mortality were 1.2% for Lobectomy, 0.68% for Wedge resection and 5.06% for Pneumonectomy.

A Kaplan-Meier Survival analysis for Lobectomies are 88.82%, 72.35% and 66.94%, for Wedge resections are 91.8%, 67.37% and 61.2%, and for Pneumonectomies are 78.41%, 44% and 25.3% at 1 year, 3 years and 5 years respectively.

## Discussion/Conclusion

We compared our results with two major European thoracic registries (STCS Bluebook 2011 and ESTS Silverbook 2014). Our in hospital mortality were 1.2% for Lobectomy vs 2.1% (SCTS Bluebook 2011) Vs 2.3% (ESTS Silverbook 2014), 0.68% for Wedge resection vs 0.7% (SCTS Bluebook 2011) Vs 1.7% (ESTS Silverbook 2014) and 5.06%for Pneumonectomy vs 6.5% (SCTS Bluebook 2011) Vs 6.3% (ESTS Silverbook 2014).

Our limited study has demonstrated a comparable and superior outcome proves that Cardiothoracic Surgeons can deliver safe and effective thoracic surgical service.

